# *In situ* single-molecule investigations of the impacts of biochemical perturbations on conformational intermediates of monomeric α-synuclein

**DOI:** 10.1063/5.0188714

**Published:** 2024-03-01

**Authors:** Wenmao Huang, Jingzhun Liu, Shimin Le, Mingxi Yao, Yi Shi, Jie Yan

**Affiliations:** 1 Department of Physics, National University of Singapore, Singapore 117542; 2 Department of Chemical Biology, School of Chemistry and Chemical Engineering, Shanghai Jiao Tong University, Shanghai 200240, China; 3 Mechanobiology Institute, National University of Singapore, Singapore 117411; 4 Research Institute for Biomimetics and Soft Matter, Fujian Provincial Key Lab for Soft Functional Materials Research, Department of Physics, Xiamen University, Xiamen 361005, China; 5 Department of Biomedical Engineering, Southern University of Science and Technology, Shenzhen 518055, China; 6 School of Chemistry and Molecular Engineering, East China Normal University, Shanghai 200241, China; 7 Centre for Bioimaging Sciences, National University of Singapore, Singapore 117546

## Abstract

α-Synuclein aggregation is a common trait in synucleinopathies, including Parkinson's disease. Being an unstructured protein, α-synuclein exists in several distinct conformational intermediates, contributing to both its function and pathogenesis. However, the regulation of these monomer conformations by biochemical factors and potential drugs has remained elusive. In this study, we devised an *in situ* single-molecule manipulation approach to pinpoint kinetically stable conformational intermediates of monomeric α-synuclein and explore the effects of various biochemical factors and drugs. We uncovered a partially folded conformation located in the non-amyloid-β component (NAC) region of monomeric α-synuclein, which is regulated by a preNAC region. This conformational intermediate is sensitive to biochemical perturbations and small-molecule drugs that influencing α-synuclein's aggregation tendency. Our findings reveal that this partially folded intermediate may play a role in α-synuclein aggregation, offering fresh perspectives for potential treatments aimed at the initial stage of higher-order α-synuclein aggregation. The single-molecule approach developed here can be broadly applied to the study of disease-related intrinsically disordered proteins.

## INTRODUCTION

Parkinson's disease (PD) is a prevalent neurodegenerative disorder that affects approximately 10 × 10^6^ people around the world.^1,2^ The cytopathological hallmarks of PD and other synucleinopathies include Lewy bodies and Lewy neurites, which contain aggregates of α-synuclein, a protein abundantly expressed in the central nervous system.^3–5^ Several mutations in the α-synuclein encoding gene (SNCA) have been identified as key factors in the pathogenesis of PD, which modulate the aggregation propensity of α-synuclein.^6–9^ Emerging evidence suggests that the misfolded α-synuclein monomers, aggregated cytotoxic oligomers, and small amyloid fibrils that act as prion-like seeds for aggregate propagation are the key pathological factors in synucleinopathies.^5,10–16^

**FIG. 1.**
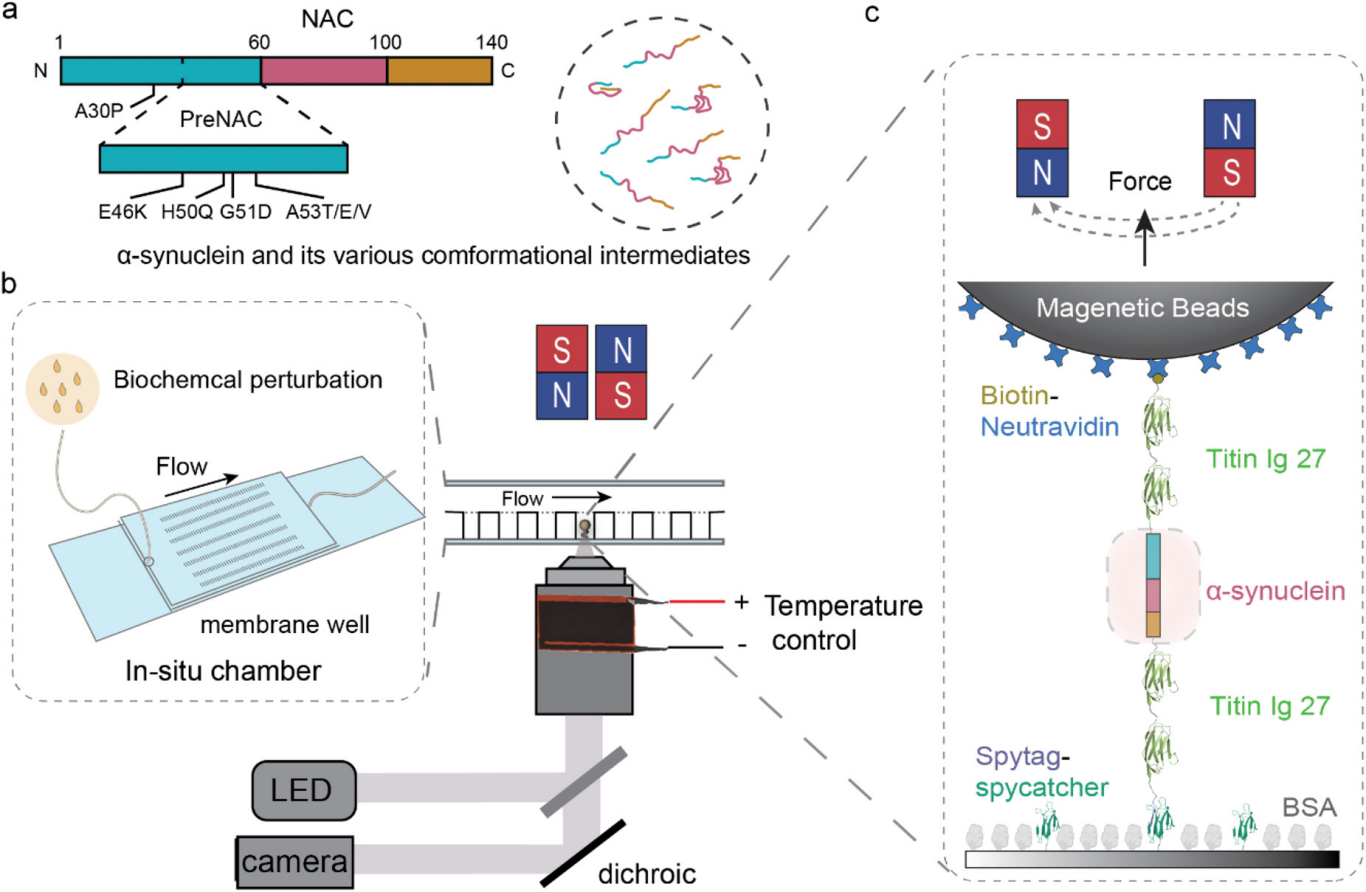
*In situ* magnetic-tweezer-based single-molecule approach. (a) Illustrated sequence map of wild-type α-synuclein (1 − 140 a.a.), alongside potential conformational intermediates of α-synuclein. (b) Schematic diagram of the experimental setup for *in situ* magnetic-tweezer-based single-molecule manipulation, comprising a magnetic tweezer for precise mechanical manipulation of proteins in the piconewton range with nanometer resolution, an *in situ* buffer exchange chamber with membrane wells, and a temperature control system. (c) Elaborate schematics showcasing that the recombinant monomeric α-synuclein was tethered to the glass substrate and the superparamagnetic beads through specific SpyTag–SpyCatcher chemistry and biotin–neutravidin bond, respectively.

α-Synuclein is an intrinsic disordered protein (IDP) that has long been considered as a soluble monomer.^17–19^ This 140-amino acids protein comprises three distinct regions [Fig. 1(a)]: a positively charged N-terminal region (amino acid, a. a. 1–60), a non-amyloid- β component (NAC) region (a. a. 61–100), and a negatively charged intrinsic disordered C-terminal region (a. a. 101–140).^17,18^ We note that in our work, we expand the previously defined NAC region^18^ from 61–95 to 61–100, which constitutes the major core structure of α-synuclein amyloid fibril.^20–24^ The hydrophobic core of α-synuclein aggregates is formed by the NAC region,^25,26^ whose aggregation propensity is regulated by an N-terminus subregion (preNAC, a. a. 36–60, containing most of the mutations) adjacent to the NAC region.^26,27^ The charged N-terminal and C-terminal regions exhibit transient long-range intra-molecule interactions as well as interactions with the cytoplasm components,^17^ which are believed to help shield the hydrophobic NAC core from exposure to the cytoplasm and keep the monomer isoform *in vivo*.^17,28,29^ Upon binding to the lipid membrane, the N-terminal and NAC regions can form an α-helical structure,^30^ which represents the only solved monomeric structure of α-synuclein to date.^18^

Cryo-electron microscopy has fully characterized the fibril-like structures of α-synuclein, revealing common fibril polymorphs featuring a β-rich kernel structure comprising the preNAC and NAC regions (36–100).^20–22,24,31^ During the propagation process from soluble monomers to fibril-like aggregates, cytotoxic^11,12,14,32^ or protective^11,33^ oligomers have been proposed to exist. At the monomer level, increasing evidence suggests that the disordered monomer is highly dynamic with versatile conformational structures [Fig. 1(a)], which can be influenced by various physiologically and pathologically relevant conditions.^17,18,28,29,34–36^ In-depth *in vitro* or *in vivo* nuclear magnetic resonance (NMR) experiments have systematically studied the inter- or intramolecular interactions of α-synuclein, including the effects of lipid vesicle binding, N-terminal and C-terminal region regulation, chaperone binding, post-translational modifications, and mutations.^17,18,27–29,35,37^

Importantly, circular dichroism (CD) and NMR spectral studies have suggested the presence of partially folded intermediates of α-synuclein with varying aggregation propensity.^17,18,28,34^ Some of the intramolecular interactions have reported to suppress the aggregation of α-synuclein, where the exposed NAC is shielded by long-range intramolecular interactions involving the NAC and C terminus.^38,39^ Consistently, several single-molecule studies have reported multiple conformational intermediates in monomeric α-synuclein.^11,36,40–42^ In particular, an optical tweezers-based study reported that the majority (85%) of the sampled α-synuclein monomers lacked any stable or metastable structures, while the remaining 15% contained diverse partially folded structures that can unfold at forces within 15 pN^36^.

Building on previous reports of intermediates in monomeric α-synuclein, along with their potential connection to aggregation, we embarked on an investigation into the regulation of conformational intermediates in monomeric α-synuclein. We utilized a magnetic-tweezer-based single-molecule manipulation approach for this purpose, where the potential intermediates in monomeric α-synuclein were recognized based on their unique mechanical signatures. We then applied *in situ* perturbations to examine the effects on these intermediates. In line with the previous research, we identified multiple conformational intermediates. In addition to a primary conformational intermediate, characterized by a disordered and randomly coiled peptide conformation, we uncovered a previously unknown major intermediate that features a partially folded element in the NAC region with a stable structure. This intermediate, henceforth referred to as the PF intermediate, is regulated by the preNAC region. Intriguingly, and potentially significantly, the PF intermediate can be finely adjusted through biochemical perturbations and specific potential drugs known to influence higher-order α-synuclein aggregation.

## RESULTS

### *In situ* single-molecule manipulation approach

To detect potential conformational intermediates in monomeric α-synuclein, we developed a magnetic-tweezer-based single-molecule approach to probe its mechanical signatures. Specifically, α-synuclein was spanned in four well-characterized titin I27 Ig domains,^43^ which served as a molecular spacer and mechanical fingerprint for single-molecule tethers (supplementary material note 1). It has been reported that spanning α-synuclein between structural domains can effectively slow down higher-order oligomerization of α-synuclein.^44^ Consistently, we confirmed that the spanning I27 domains in our study prevented aggregation or oligomerization of the recombinant protein constructs (supplementary material Fig. 1). Moreover, we found that the recombinant proteins remained as monomers for up to five days (supplementary material Fig. 1, supplementary material Note 1).

The mechanical signatures of potential distinct conformational intermediates of α-synuclein could be manifested as incremental extension changes resulting from abrupt conformational shifts, along with the corresponding forces when these transitions occur. To examine how biochemical factors and temperature impact the conceivable conformational intermediates of monomeric α-synuclein, we employed a disturbance-free membrane-well system that mitigates unwanted flow-induced stretching of the single-molecule tether during buffer exchange^45^ [Fig. 1(b)]. This system is crucial for the *in situ* analysis of the effects of various biochemical perturbation. Furthermore, to investigate the potential influence of temperature on conformational intermediates, we employed an objective-heating system to precisely regulate the temperature within the sample chamber [Fig. 1(b)].

The recombinant protein construct, which was fused with an N-terminal biotinylated AviTag and a C-terminal SpyTag, was anchored between a Neutravidin-coated superparamagnetic microbead (2.8 *μ*m in diameter, M270, Invitrogen) and a SpyCatcher-coated coverslip glass surface [Fig. 1(c)]. Subsequently, this tether was subjected to cycles of force-increase and decrease scans between 1.5 ± 0.1 and 100 ± 10 pN at constant loading rates (see the supplementary methods). We would like to note that the transition forces are associated with a 10% uncertainty arising from our force-calibration method.^46^ During these force scans, the height of the end-attached superparamagnetic microbead was continuously monitored. We note that when there is a change in force, the corresponding change in bead height is influenced by two factors: the change in the extension of the tethered molecule and the rotation of the bead due to torque rebalancing.^46^ Conversely, bead height changes at a constant force or stepwise bead height changes during force loading represent the extension change of the molecule. The specific mechanical responses of a single tethered α-synuclein molecule were ensured by the signatures of mechanical unfolding of the four I27 domains at forces above 80 pN (supplementary material Fig. 2).

**FIG. 2.**
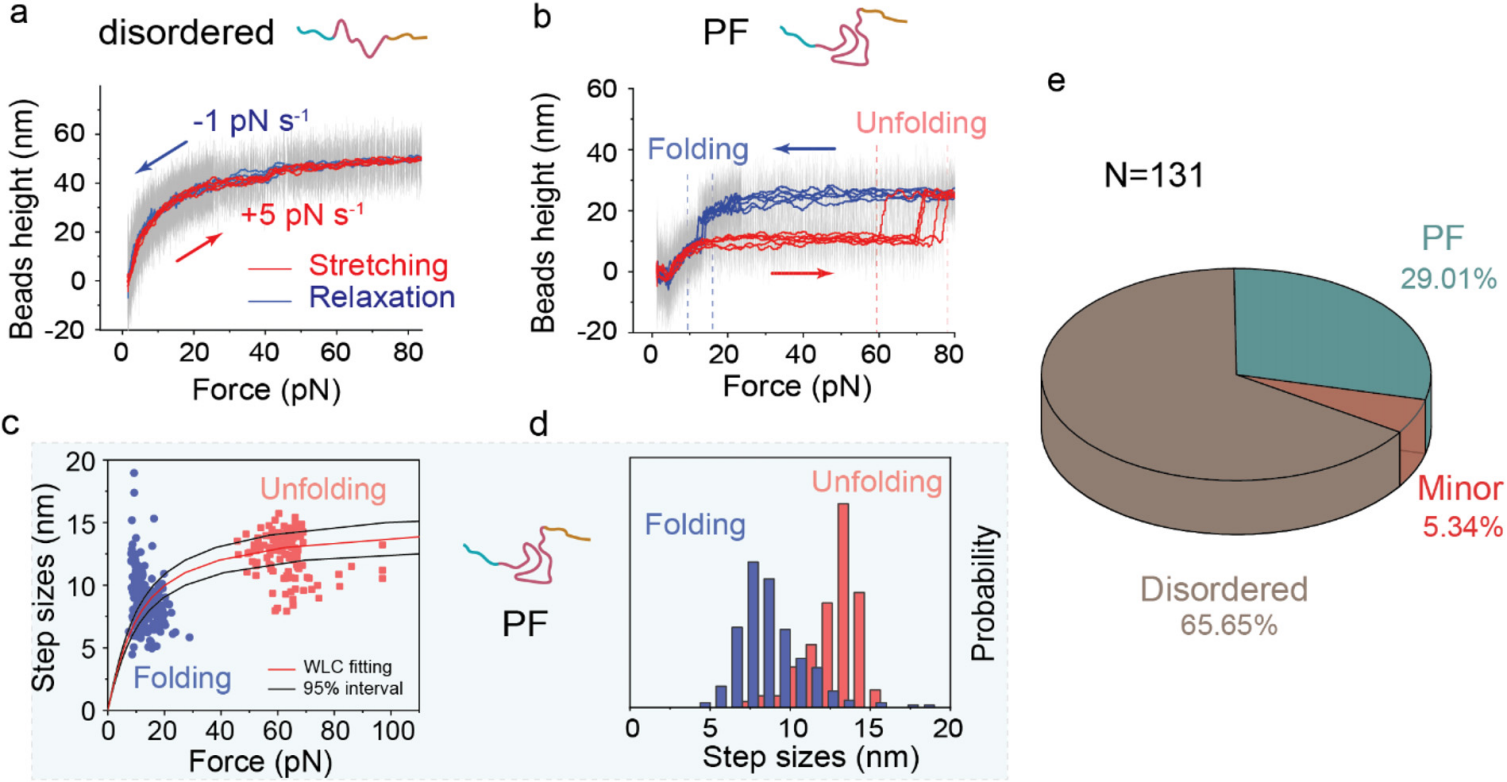
Two major conformational intermediates of monomeric α-synuclein. (a) Typical force−bead height curves of a disordered monomeric α-synuclein during force-increase scan (red, loading rate of 5 pN s^−1^) and subsequent force-decrease scan (blue, loading rate of −1 pN s^−1^). (b) Typical force−bead height curves of partially folded (PF) α-synuclein during force-increase and force-decrease scans at the same corresponding loading rates as in (a). (c) Unfolding and refolding force-step size distribution of PF α-synuclein at loading rates of 5 pN s^−1^ (red dots) and −1 pN s^−1^ (blue dots), respectively. A worm-like chain model was used to fit the data (red line), and the optimal fitting persistence length (A) and contour length (Lc) were obtained as A = 0.50 ± 0.05 and Lc = 15.50 ± 1.50 nm. ± indicates S.E. (d) Normalized unfolding and refolding step size distributions of PF α-synuclein, respectively. The data in (c) and (d) are from 124 (unfolding) and 268 (refolding) events, which were collected from more than 30 independent α-synuclein tethers. (e) Conformation fractions of monomeric α-synuclein (N = 131 molecules) based on their distinct mechanical signatures (supplementary material Fig. 8).

### Identification of conformational intermediates of monomeric α-synuclein

We demonstrate a significant conformational species (disordered) of monomeric α-synuclein, as evidenced by a smooth force-bead height curve that lacks stepwise structural transitions over the experimental timescale at specific loading rates [Fig. 2(a), supplementary material Fig. 2(a)]. Due to the aforementioned bead rotation, the smooth force-bead height curve does not represent the force-extension curve of the tethered molecule. Consequently, it is not possible to estimate the contour length of the underlying disordered polypeptide chain. However, our control experiments suggest that the observed smooth force-bead height curves can be explained as a random, unstructured coiled conformation of α-synuclein (supplementary material Note 2 and supplementary material Fig. 3). This aligns with the anticipated presence of such an unstructured coiled conformation given the inherently disordered nature of this protein and is consistent with the observation from previous studies.

**FIG. 3.**
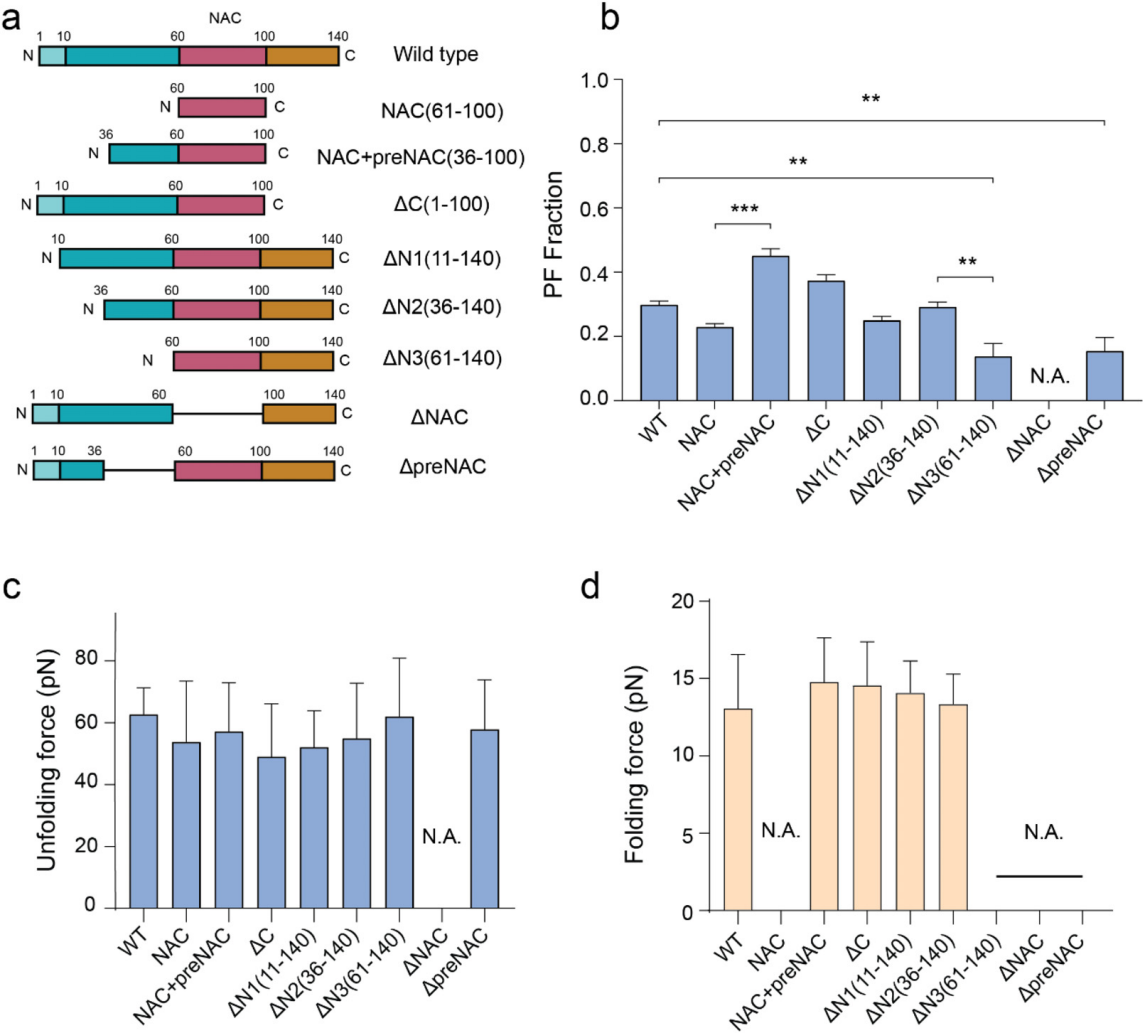
Effects of truncations of α-synuclein. (a) Illustrated domain maps of wild-type and truncated α-synucleins. (b) PF population fractions of wild-type α-synuclein (WT) and various truncations based on the unfolding signature of the PF species. The numbers of tested molecules are 131, 35, 20, 74, 60, 48, 51, 47, and 52 for WT, NAC, NAC + preNAC, ΔC, ΔN1, ΔN2, ΔN3, ΔNAC, and ΔpreNAC, respectively. Error bars indicate mean ± S.E.M. (c) The average unfolding forces during force-increase scans at a loading rate of 5 pN s^−1^. The numbers of unfolding events are: 124, 153, 193, 199, 161, 237, 16, 0, and 21 for WT, NAC, NAC + preNAC, ΔC, ΔN1, ΔN2, ΔN3, ΔNAC, and ΔpreNAC, respectively. (d) The average folding forces during force-decrease scans at a loading rate of −1 pN s^−1^. The numbers of folding events are: 268, 0, 76, 55, 98, 89, 0, 0, and 0 for WT, NAC, NAC + preNAC, ΔC, ΔN1, ΔN2, ΔN3, ΔNAC, and ΔpreNAC, respectively. Error bars in Fig. 3(c) and 3(d) indicate mean ± S.D. N.A. means not applicable (no PF conformation is observed). ^**^p < 0.01; ^***^p < 0.001 (95% of confidence intervals). p values were determined using an unpaired two-tailed Student's t-test.

Unexpectedly and intriguingly, the experiments also revealed the existence of another major conformational intermediate, i.e., the PF intermediate, featured with a partially folded element. During force-increase scans at a loading rate of 5 pN $s^{-1}$, this conformational species exhibited a distinct unfolding step at approximately 60 pN associated with a step size of around 14 nm. Subsequently, when force was reduced at a loading rate of −1 pN $s^{-1}$ to around 15 pN, this species could refold, resulting in a step size of roughly 8 nm [Figs. 2(b)–2(d) and supplementary material Fig. 2(b)]. The unfolding and refolding step sizes were indicative of the release or absorption of a peptide of 41 ± 4 amino acids (a.a.) under force [Fig. 2(d) and supplementary material Fig. 4]. This uncertainty contributes to the spread of the step size histograms, similar to the level observed in our previous studies of well-defined structural domains, such as titin I27^44^ and α-actinin spectrin repeats.^47^ This peptide (41 ± 4 a.a.) is much shorter than α-synuclein's total amino acid count (140 a.a.), implying that the unfolding and refolding signatures are from partially folded element in the PF intermediate. Additionally, the PF intermediate was detected in control experiments using the α-synuclein protein without the I27 domains, which rules out the possibility that it is induced by the spanning I27 domains (supplementary material Fig. 5 and supplementary material Note 1].

**FIG. 4.**
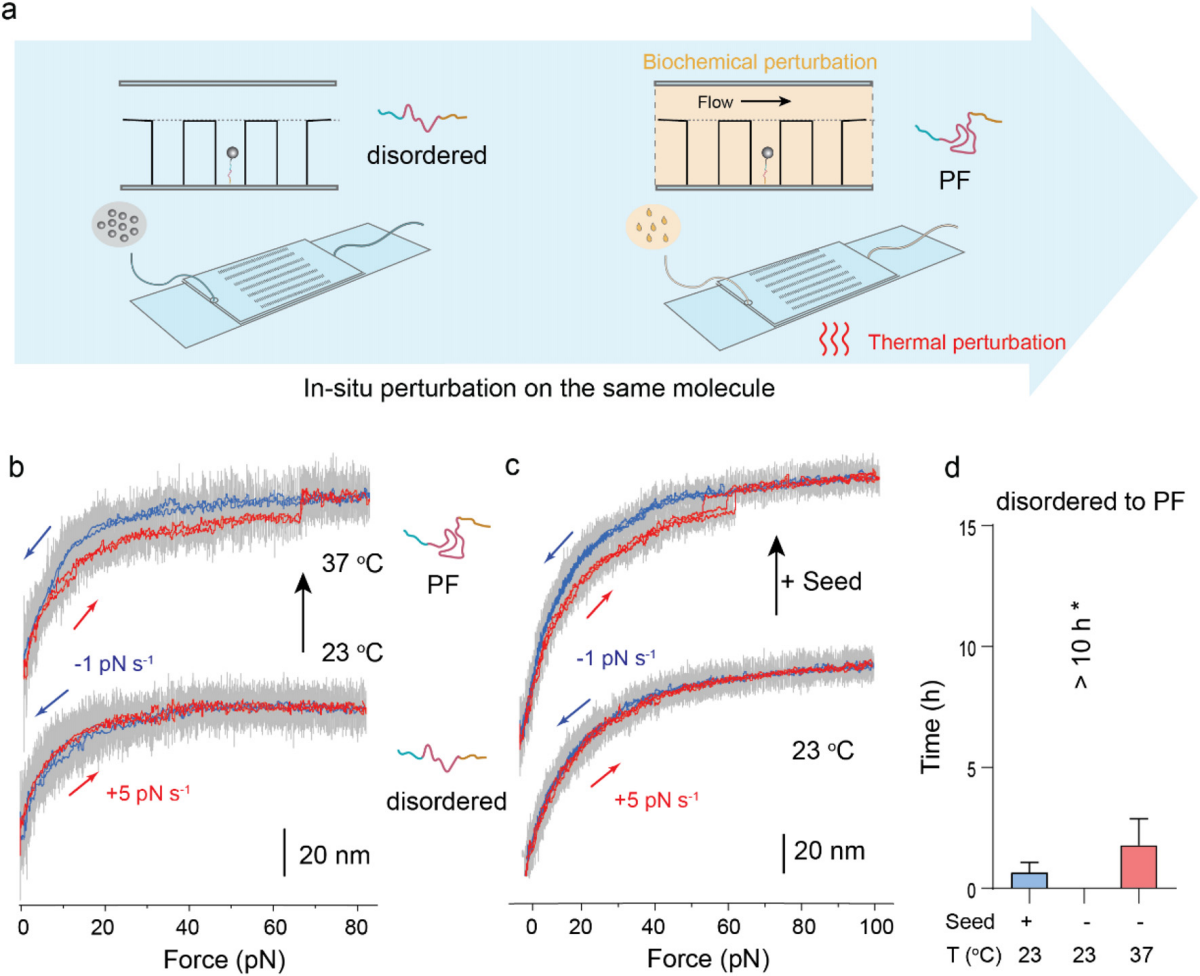
*In situ* perturbations of the disordered-to-PF conversion. (a) Schematic illustration of the *in situ* single-molecule experiments. Single disordered α-synuclein was identified at 23 °C (left panel), and the seed (small α-synuclein aggregates) was introduced or the temperature was increased to 37 °C (right panel). (b) Representative force-bead height traces of the disordered α-synuclein at increasing temperatures from 23 °C (bottom) to 37 °C (top). Similar results were observed in three independent tethered proteins. (c) Representative force-bead height traces of the α-synuclein before (bottom) and after (top) flowing in 10 nM α-synuclein seed at 23 °C. Similar results were observed in four independent tethered proteins. (d) Average times required for the conversion from the disordered to PF conformations of α-synuclein under the corresponding *in situ* perturbations. Error bars indicate mean ± S.E.M. ^*^For comparison, the disordered-to-PF transition at 23 °C without seed was not observed over 10 h.

**FIG. 5.**
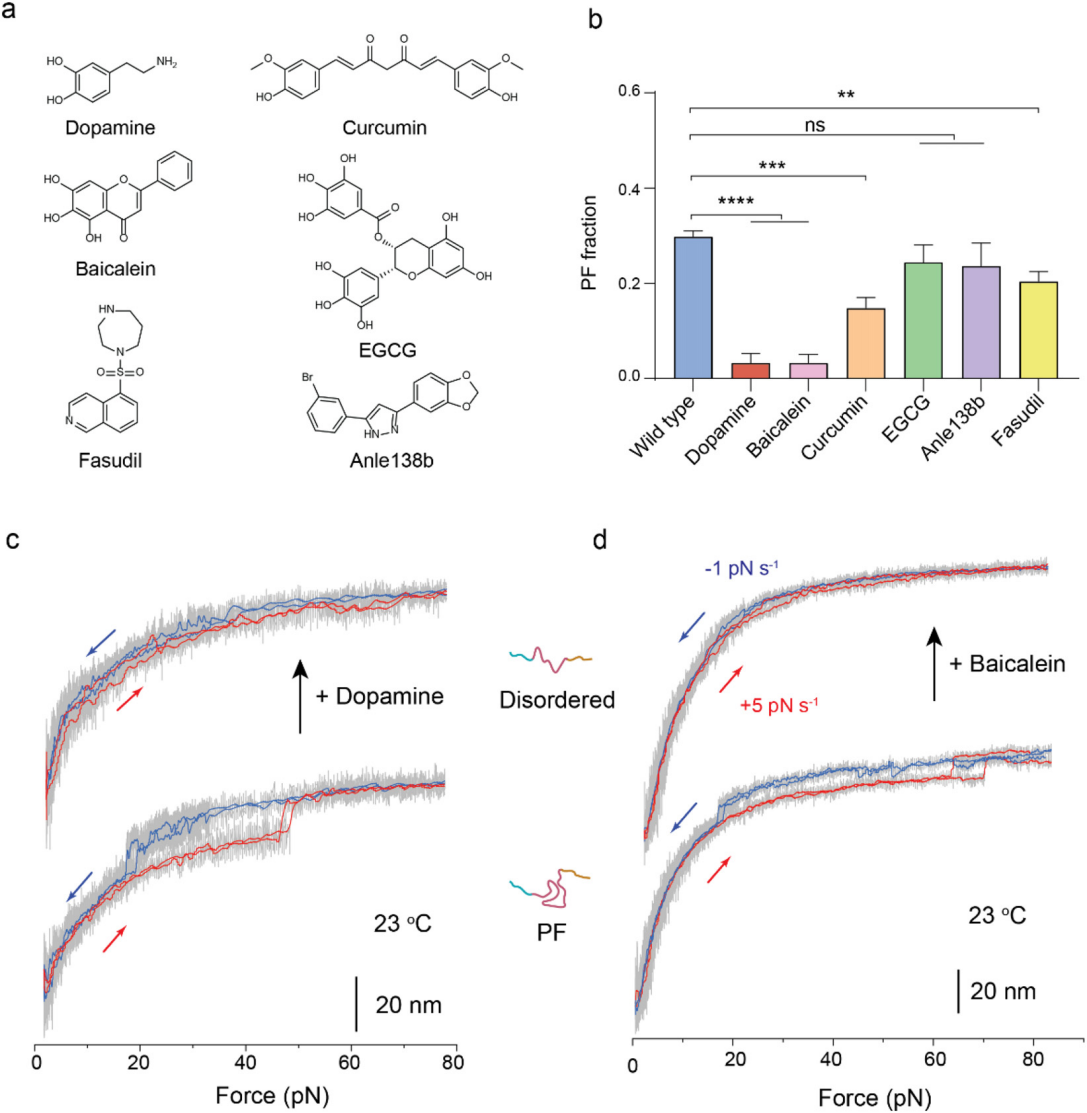
Effects of small-molecule drugs. (a) Chemical structures of six small-molecule drugs reported to inhibit the *in vitro* aggregation of α-synulcein. (b) PF fraction of wild-type and small-molecule pretreated α-synulcein. The total molecule numbers are N = 131, 62, 61, 61, 45, 55, and 54 for wild type, six small-molecule (dopamine, baicalein, curcumin, EGCG, anle138b, and fasudil) treated α-synulcein, respectively. Error bars indicate mean ± S.E.M. ns: p > 0.05; ^**^p < 0.01; ^***^p < 0.001; and ^****^p < 0.0001 (95% of confidence intervals). p values were determined by unpaired two-tailed Student's t-test. (c) and (d) *In situ* analysis of the PF-to-disordered transition by dopamine and baicalein. (c) Representative force−bead height curves of a PF α-synuclein before (bottom) and after (top) introducing 0.1 mM dopamine solution. Similar results were repeated on three independent α-synucleins. (d) Representative force-bead height curves of a PF α-synuclein before (bottom) and after (top) introducing 0.1 mM baicalein solution. Similar results were observed on five independent tethered proteins.

The partially folded element in the PF intermediate exhibits a high level of mechanical stability. It required high forces (typically > 50 pN) for unfolding at various force-increase loading rates (0.5 − 5 pN s^−1^) (supplementary material Fig. 4). It also survived substantial lifetime at constant high forces [40–60 pN, supplementary material Fig. 6, supplementary material Note 3 for force-jump experiments]. Over 60% of the unfolded intermediate rapidly refolds at forces of around 15 pN at various force-decrease loading rates (−2.0 to −0.1 pN s^−1^) (supplementary material Fig. 4). The rapid refolding of the partially folded intermediate is surprising and noteworthy, as it was not observed in disordered conformations. This implies that the unfolded intermediate's conformation differs from that of entirely disordered species and may lead to another intermediate that can refold back when forces are reduced to 15 pN. Although the unfolded PF species' conformation remains unclear, the outcome suggests that it may be a force-resistant structural intermediate that requires further investigation. Additionally, we found no conversion between the disordered and PF intermediates at 23 °C within our experimental timescale (tens of hours), indicating that they represent local energy minima separated by a high free energy barrier. Based on these observations, we note that the stable partially folded intermediate in the PF species is different from the intermediates reported in previous single-molecule manipulation studies.^11,36,40–42,48^

In addition to the primary conformational species (disordered: approximately 65%; PF: approximately 30%), we also identified a minor conformational species (roughly 5%) consisting of various partially folded intermediates. Each of these intermediates exhibits distinct mechanical signatures based on transition forces and step sizes (supplementary material Note 4 and supplementary material Fig. 7). The population fractions of these conformational species [Fig. 2(e) and supplementary material Fig. 8) have been quantified using the mechanical signatures from randomly selected tethered protein constructs, which represent the population fractions of different α-synuclein conformational species coexisting in the solution.

### The partially folded element situates in NAC and is regulated by preNAC

To locate the region of the partially folded intermediate in the PF species and the regions that may influence its stability, we carried out single-molecule experiments on various truncated α-synuclein constructs [Fig. 3(a)], including NAC (a.a. 61 − 100), NAC + preNAC (36–100), ΔC (1–100), and three N-terminal truncations ΔN1 (11–140), ΔN2 (36–140), ΔN3 (61–140), as well as two truncates termed as ΔNAC, ΔpreNAC without NAC, or preNAC region. Like wild-type α-synuclein, all truncated α-synuclein variants were spanned between two I27 domains at each side to maintain their monomeric conformations (See the supplementary material for details of protein construct sequences).

The primary findings from single-molecule force scans are summarized in Fig. 3(b) and supplementary material Fig. 9. The complete disappearance of the characteristic unfolding/refolding signatures of PF was observed in the NAC truncation, indicating that the partially folded intermediate is in the NAC region. Apart from the ΔNAC construct, all other truncated variants retained the characteristic unfolding signature of the partially folded intermediate.

The unfolding and refolding signatures of the PF species for NAC + preNAC, ΔC, ΔN1, and ΔN2, were found to be identical to those of wild-type α-synuclein [Figs. 3(c) and 3(d), supplementary material Figs. 10 and 11]. Notably, in the absence of the preNAC region in NAC, ΔN3, and ΔpreNAC, the unfolding signature was preserved, whereas the refolding only occurred at ∼1.5 pN at a loading rate of −1 pN s^−1^ [Fig. 3(d), supplementary material Figs. 10 and 11]. The refolding of the unfolded structure undergoes either the rapid refolding pathway characterized by the ∼15 pN refolding forces or the slow refolding pathway at ∼1.5 pN [supplementary material Figs. 11(c)]. The presence of the preNAC region was found to be essential to the rapid refolding pathway, while its absence led to a much slower refolding of the unfolded PF structure [supplementary material Figs. 11(d)−11(f)]. These results suggest that the preNAC region modulates the refolding kinetics of the PF's partially folded intermediate after its unfolding.

Based on these observations, we conclude that (1) the NAC region bears the identified partially folded element in the PF intermediate and (2) the preNAC region facilitates its folding, thus contributing to the regulation of the partially folded intermediate. We speculate that preNAC might interact with the unfolded conformation of the element, promoting folding along a specific pathway. Notably, prior studies have reported that the deletion of preNAC significantly suppresses the aggregation of α-synuclein,^27^ while several familial mutations of early onset PD were located in the preNAC region.^6–9^ We reason that these mutations may influence the preNAC-mediated regulation of the PF species, which is our ongoing study.

### Biochemical perturbations on PF intermediates

Given that the identified PF intermediate was not reported in previous studies and that it represents a major form of the monomeric α-synuclein, we investigated the potential effects of two known aggregation promoting factors, temperature, and pion-like seeds, on the formation of the PF intermediate.

It is known that the preparation of α-synuclein can have a pronounced effect on its aggregation properties.^49^ In order to ensure identical initial conditions for the investigations, the monomeric α-synuclein proteins were fully unfolded using 6 molars (M) guanidine hydrochloride (GuHCl) and 8 M urea, and subsequently diluted and flowed into the *in situ* sample chamber to form single-molecule tethers. Treatment with 6 M GuHCl and 8 M urea resulted in a dramatically decreased fraction of the PF intermediate to a negligible level within hours after the diluted denaturant solution was removed from the chamber, leaving the disordered conformation as the predominant species (supplementary material Note 5 and supplementary material Fig. 12). This finding is consistent with the ability of the selected denaturants (GuHCl and urea) to fully convert monomeric α-synuclein into an unstructured conformation, regardless of its initial state following protein expression and purification.

To investigate the effect of temperature, a tethered α-synuclein protein in the disordered conformation was identified at 23 °C and *in situ* subject to a temperature increase to 37 °C within 10 min [Fig. 4(a)]. The conversion to the PF intermediate of the same α-synuclein molecule was observed within hours at 37 °C [Fig. 4(b), supplementary material Fig. 13], while such conversion was not observed within 10 h at 23 °C. This result indicates that higher temperatures increase the rate of the formation of the PF intermediate converted from an initially disordered conformation. In the experiment to test the effect of prion-like seed perturbation, a tethered α-synuclein protein in the disordered conformation was identified at 23 °C and then *in situ* exposed to a solution containing prion-like seeds (∼10 nM, equivalent to the molar concentration of monomer) at the same temperature [Fig. 4(a)]. The conversion of the same α-synuclein molecule to the PF intermediate conformation was also observed within hours after the introduction of seeds [Fig. 4(c), supplementary material Fig. 13]. In contrast, without the seeds, such conversion was not observed up to tens of hours at 23 °C [Fig. 4(d)].

In the above-mentioned *in situ* experiments, the conversion from the disordered species to the PF intermediate was based on similar unfolding forces and step sizes to those observed in Fig. 2. However, we observed a difference that the previously noted refolding of the unfolded PF at forces around 15 pN is absent in both cases (higher temperature or seed treatment). This could indicate an effect from temperature and seed on its refolding pathway. In addition, we found that the disordered α-synuclein could also be converted to PF intermediate by *in vitro* pretreatment experiment, where the denatured unstructured proteins in solution were treated by seeds or incubated at different temperatures, prior to single-molecule testing [supplementary material Note 5, supplementary material Fig. 12].

Together, the *in situ* perturbation single-molecule mechanical assays have shown that the PF intermediate of the monomeric α-synuclein molecules is positively modulated by increased temperature and prion-like seeds.

### Two potent small-molecule inhibitors of PF

Several natural or synthesized small molecules have been demonstrated to suppress the formation of α-synuclein fibrils, and some of these compounds are potential therapeutic agents for neurodegeneration diseases associated with α-synuclein aggregation.^50–56^ Multiple mechanisms have been proposed to explain the activity of these small molecules, including destabilization of the monomer–monomer interface within α-synulcein fibrils and modification of the conformation of α-synuclein monomers to discourage aggregation.^50,52,53,56,57^ Here, we explore the possibility that certain small molecules may affect the PF intermediate of monomeric α-synulcein identified in this study.

The recombinant α-synuclein proteins were pretreated with six small molecules [Fig. 5(a)] at a concentration of 0.1 mM for 24 h at 23 °C, without prior exposure to a denaturant. After removing the small molecules from the single-molecule experimental flow chamber, we assessed the initial population fraction of the PF intermediates using magnetic tweezers. Four of the six small molecules, namely, dopamine, baicalein, curcumin, and fasudil, showed significant suppressive effects on the PF species. Dopamine and baicalein exhibited the most potent inhibitory effects [Fig. 5(b)]. As α-synulcein monomers were not pretreated with a denaturant, it suggests that the potent inhibitory effects of dopamine and baicalein are mediated by direct binding and catalyzing conversions from the PF species to other conformations.

The effects of dopamine and baicalein were further confirmed with *in situ* single-molecule experiments on the same tethers. A tethered α-synuclein protein in the PF intermediate conformation was identified at 23 °C and then *in situ* exposed to a solution containing 0.1 mM dopamine or baicalein at the same temperature. The conversion of the same α-synuclein molecule to from the PF to disordered conformation was observed within 30 min after the introduction of the drugs [Figs. 5(c) and 5(d), supplementary material Fig. 13].

Previous studies have demonstrated that dopamine can directly bind α-synuclein and might alter its conformation,^52,58^ yet details of the inhibitor's binding site and whether they could bind to the unfolded state^59^ remain unclear. Based on our observation that dopamine and baicalein exhibit potent inhibitory activity against the PF conformation, we reason that dopamine and baicalein may bind to critical residues in pre-NAC/NAC regions that suppresses the PF conformation. PD has been linked to low or declining levels of dopamine in the brain.^58,60^ Our results provide a possible mechanistic understanding, as decreased dopamine level may lead to increased PF species that could promote α-synuclein aggregation.

## DISCUSSIONS

In this study, we have developed a single-molecule manipulation approach to delve into the distinct conformational states within monomeric α-synuclein molecules. More importantly, we have explored the modulation of the *in situ* conversion among these conformational intermediates under various biochemical conditions. This single-molecule approach is integrated with conventional bulk assays, providing a comprehensive comprehension of the diverse conformational landscape of monomeric α-synuclein.

The method we propose involves the generation of monomeric α-synuclein molecules by spanning them between two titin I27 immunoglobulin domains situated at each end. Biochemical assays have substantiated the ability of these I27 domains to sustain the monomeric state of α-synuclein for a duration of at least 5 days. Additionally, our methodology facilitates the discrimination of multiple conformational states of monomeric α-synuclein, discerned by unique mechanical signatures. Specifically, these signatures encompass unfolding and refolding forces, as well as transition step sizes observed during cycles of incremental and decremental force scans, quantified using a magnetic tweezers setup. The *in situ* conversion of monomeric α-synuclein is facilitated through a temperature control system paired with a flow disturbance-free rapid solution exchange technology. Collectively, these techniques offer a robust, magnetic tweezers-based single-molecule platform ideal for investigating the *in situ* conversions of various conformational states of monomeric α-synuclein, and potentially other intrinsically disordered proteins.

One significant discovery from this study is the unveiling of a previously undiscovered conformational state within monomeric α-synuclein, referred to as the PF intermediate. Distinguished by a stable, partially folded structure, the PF intermediate exhibits distinct mechanical traits in the NAC region—a domain acknowledged for composing the hydrophobic core of α-synuclein fibrils. The appearance of the PF intermediate is intricately modulated by the preNAC region, which facilitates its reformation following the unfolding of the partially folded element within the PF intermediate. Intriguingly, increased temperatures and the presence of prion-like seeds were found to accelerate the transformation from the initial disordered conformation to the PF intermediate, as unveiled through *in situ* perturbations in single-molecule investigations. In contrast, dopamine and baicalein were observed to expedite the reverse conversion from the PF intermediate back to the original disordered conformation. These findings serve to enrich our comprehension of the intricate conformational landscape of monomeric α-synuclein (supplementary material Fig. 14). Moreover, it would be intriguing for future studies to investigate whether the factors influencing the promotion or inhibition of the PF intermediate demonstrate additive effects when working together. It is worth noting that previous single-molecule assays have missed the identification of the PF intermediate. This could be attributed to insufficient forces (<40 pN) that could not cause its unfolding in an optical-tweezers-based study, or non-optimally positioned fluorophores that were not sensitive enough to detect the extension difference between the disordered and the PF species in single-molecule FRET assays.^40,42^

The crucial initial stage leading to α-synuclein aggregation remains to be determined. Various studies have proposed that a key step in pathogenic aggregation involves oligomers.^12,14,15,32,61–64^ Additionally, other research suggests that the monomeric conformational structures of α-synuclein may play a critical role in the early stages of aggregation.^17,18,28,41,65^ The strong correlation between the occurrence of the identified PF intermediate and factors that are known to regulate the aggregation propensity of α-synuclein suggest a positive relation of the PF intermediate to the aggregation propensity of α-synuclein. Since α-synuclein aggregation requires hydrophobic residues in the NAC region,^38,39^ in the PF intermediate the necessary hydrophobic residues required for aggregation should remain accessible. Once incorporated into the aggregated state, further conformational changes of α-synuclein proteins can be expected.

Finally, we would like to emphasize that this study is limited to *in vitro* experiments solely based on mechanical signatures. As a result, it does not offer insights into the potential structure of this intermediate species. Furthermore, while the findings reveal a positive correlation between the intermediate in the PF species and α-synuclein aggregation, its actual roles in α-synuclein aggregation remain to be determined in future studies. While the force-based approach is a powerful tool to probe different intermediate conformations based their different mechanical responses, it does not imply that α-synuclein monomers are under mechanical stretching in its physiologically relevant conditions. Another limitation of our study is that due to the bead rotation during force change, we cannot determine the accurate force-extension curve of the disordered conformational intermediate. Therefore, the specific characteristics, such as its flexibility, of the disordered conformational intermediate remain incompletely understood. We note that the force-extension curves lacking steps within the experimental range does not precisely equal to disordered conformation. They could alternatively be explained by structures that unfold at very low forces that cannot be probed by the measurement, or structures with ultra-mechanical stability that do not unfold during our stretching processes.

## METHODS

### Reagents and materials

(3-aminopropyl)triethoxysilane (APTES), glutaraldehyde solution (70% in H_2_O), dithiothreitol (DTT), bovine serum albumin (BSA), urea, guanidine hydrochloride (GuHCl), Sodium dodecyl sulfate (SDS) (−)-Epigallocatechin gallate (EGCG), curcumin from Curcuma longa (Turmeric), fasudil hydrochloride, baicalein, Anle138b, dopamine hydrochloride, thioflavin T (ThT), L-ascorbic acid, ampicillin, isopropyl β-d-1-thiogalactopyranoside (IPTG), phenylmethylsulfonyl fluoride (PMSF), biotin, mineral oil, PBS, HEPES, and Tris buffer were purchased from Sigma-Aldrich. NeutrAvidin biotin-binding protein, Dynabeads™ M-270 Epoxy beads were purchased from Thermo Scientific. Recombinant human α-synuclein protein monomer and recombinant human α-synuclein protein aggregate (seeds) were prepared following the previous method.^66^

### Gene construction, protein expression, and purification

The human α-synuclein SNCA gene (residue 1-140, Uniprot P37840) was codon-optimized and synthesized for expression in *Escherichia coli* as gBlocks gene fragments (Integrated DNA Technologies (IDT)) with suitable overhangs. The gene was fused with four tandem titin immunoglobulin domains (Ig27) of a pET151 Vector (ampicillin resistance) with 6×His-tag for purification, AviTag for biotinylation, and SpyTag using the NEB HiFi assembly strategy (New England Biolabs, MA, USA). The construction of SpyTag binding protein−SpyCatcher was referred to the previous works.^47^ Truncated variants [NAC (a.a. 61–100), NAC + preNAC (a.a. 36–100), ΔC (a.a. 1–100), ΔN1 (a.a. 11–140), ΔN2 (a.a. 36–140), ΔNAC (a.a. 1–60 + 101–140), and ΔpreNAC (a.a. 1–36 + 61–140)] were introduced using the site-directed mutagenesis kit (New England Biolabs, MA, USA). The complete sequences of all recombinant protein constructs used in this study are listed in the supplementary material (“Protein construction and sequence” section). All the α-synuclein-Ig27 fused protein constructs were expressed in *E. Coli* DE3 with biotin protein ligase (BirA) and purified using His-tag affinity column following the previous protocol.^47,67^

The full-length α-synuclein monomer (1–140) expression plasmid (pET21a-alpha-synuclein) was a gift from Michael J Fox Foundation MJFF (Addgene plasmid # 51486). The α-synuclein monomer was expressed and purified following the previous method.^20,21^ The purified α-synuclein monomer was concentrated to 500 *μ*M in fibril growth buffer (50 mM Tris, pH 7.0, 150 mM KCl, 0.02% NaN_3_) and incubated at 37 °C with agitation (900 rpm) in an incubator (Thermo Fisher). After 5 days, the fibrillar samples were diluted to a concentration of ∼100 *μ*M (equal to the monomer concentration) and ultrasonicated for 10 min (1 s on, 1 s off) on ice to yield small size of α-synuclein aggregates (seeds). More details on protein expression and purification are listed in the supplementary methods.

### Magnetic-tweezer-based single-molecule essay

Single-molecule detection on α-synuclein proteins was achieved on a homemade magnetic tweezers (MT) setup as previously described.^46,68^ To perform the experiments, the α-synuclein-I27 fused protein constructs were tethered between Neutravidin-coated magnetic beads and SpyCatcher-coated cover glass in membrane-well laminar flow *in situ* chambers. The standardized solution used for the single-molecule experiments was composed of 20 mM HEPES, 50 mM KCl, 10 mM MgCl_2_, 1% (m/m) BSA, 1 mM DTT, pH = 7.4, unless otherwise specified. Details on the preparation of the chamber and microbeads, as well as the sample preparation, magnetic tweezer setup, constant loading rate scan, force jump experiment, and force calibration, have been previously published in our studies^47,67^ and are briefly summarized in the supplementary material.

### *In situ* single-molecule experiments

In a typical *in situ* single-molecule experiment, the chamber was connected to a buffer exchange system to facilitate changes in solution condition. The tethered protein was initially scanned for several cycles to obtain its structural conformation prior to the buffer exchange. Solutions containing denaturant, seeds, or drugs, totaling 200 *μ*l (three times the chamber volume), were added to the rapid buffer exchange system and flowed through the chamber. The membrane-well based disturbance-free system would inhibit the unwanted flow stretching perturbation to the tethered molecule. After about 10 min of incubation, the system stabilized, and force scans of the α-synuclein in the new buffer condition began.

In a typical *in situ* temperature-change single-molecule experiment, the temperature of the experiment was increased from 23 to 37 °C using an objective heating system (Bioptechs) that had been pre-calibrated with a thermometer. The tethered protein was initially scanned for several cycles at 23 °C to obtain its structural conformation prior to the temperature change. The temperature was then increased to 37 °C and maintained for more than 10 min to allow for temperature equilibrium.

### Single-molecule data analysis

Raw time traces data were recorded at a sampling rate of 200 Hz. In all the figures presented in the main text and supplementary material, the time traces and bead height−force traces were smoothed using the fast Fourier transform (FFT)-smooth function of OriginPro 9.0, unless otherwise mentioned. The time resolution of our experiment is around 5 *μ*s due to the sampling rate. To determine the unfolding and refolding force and step size for α-synucleins at different force loading rates, we collected data from multiple independent tethers. The figures and legends indicate the number of unfolding/refolding events (n) and the number of tethers (N).

To measure the conformational species fraction of α-synuclein molecules, the tethered single-molecule α-synucleins were randomly selected and scanned for more than ten cycles to obtain the mechanical responses. As the different conformations were kinetically separated, the conversion between the disordered conformation and PF was not observed over the single-molecule experimental timescale (tens of hours) at 23°C. Therefore, the tethered α-synuclein conformation fractions corresponded to the population fractions of the different conformational species of α-synuclein co-existing in the solution.

## SUPPLEMENTARY MATERIAL

See the supplementary material for details on additional experimental details and protein construct sequences, supplementary methods, supplementary notes 1 − 5, supplementary material Figs. 1–14, and related references.

## Data Availability

All data generated and analyzed during this study are included in this article and are also available from the authors upon reasonable request.

## References

[1] O.-B. Tysnes and A. Storstein , “ Epidemiology of Parkinson's disease,” J. Neural Transm. 124(8), 901–905 (2017). 10.1007/s00702-017-1686-y 28150045

[2] W. Poewe , K. Seppi , C. M. Tanner *et al.*, “ Parkinson disease,” Nat. Rev. Dis. Primers 3(1), 1–21 (2017). 10.1038/nrdp.2017.13 28332488

[3] H. A. Lashuel , C. R. Overk , A. Oueslati *et al.*, “ The many faces of α-synuclein: from structure and toxicity to therapeutic target,” Nat. Rev. Neurosci. 14(1), 38–48 (2013). 10.1038/nrn3406 23254192 PMC4295774

[4] M. G. Spillantini and M. Goedert , “ The α‐synucleinopathies: Parkinson's disease, dementia with Lewy bodies, and multiple system atrophy,” Ann. N.Y. Acad. Sci. 920(1), 16–27 (2000). 10.1111/j.1749-6632.2000.tb06900.x 11193145

[5] W. Peelaerts , L. Bousset , A. Van der Perren *et al.*, “ α-Synuclein strains cause distinct synucleinopathies after local and systemic administration,” Nature 522(7556), 340–344 (2015). 10.1038/nature14547 26061766

[6] M. H. Polymeropoulos , C. Lavedan , E. Leroy *et al.*, “ Mutation in the α-synuclein gene identified in families with Parkinson's disease,” Science 276(5321), 2045–2047 (1997). 10.1126/science.276.5321.2045 9197268

[7] J. J. Zarranz , J. Alegre , J. C. Gómez‐Esteban *et al.*, “ The new mutation, E46K, of α‐synuclein causes Parkinson and Lewy body dementia,” Ann. Neurol. 55(2), 164–173 (2004). 10.1002/ana.10795 14755719

[8] P. Flagmeier , G. Meisl , M. Vendruscolo *et al.*, “ Mutations associated with familial Parkinson's disease alter the initiation and amplification steps of α-synuclein aggregation,” Proc. Natl. Acad. Sci. U. S. A. 113(37), 10328–10333 (2016). 10.1073/pnas.1604645113 27573854 PMC5027465

[9] S. Lesage , M. Anheim , F. Letournel *et al.*, “ G51D α‐synuclein mutation causes a novel Parkinsonian–pyramidal syndrome,” Ann. Neurol. 73(4), 459–471 (2013). 10.1002/ana.23894 23526723

[10] S. Mehra , S. Sahay , and S. K. Maji , “ α-Synuclein misfolding and aggregation: Implications in Parkinson's disease pathogenesis,” Biochim. Biophys. Acta 1867(10), 890–908 (2019). 10.1016/j.bbapap.2019.03.001 30853581

[11] N. Cremades , S. I. Cohen , E. Deas *et al.*, “ Direct observation of the interconversion of normal and toxic forms of α-synuclein,” Cell 149(5), 1048–1059 (2012). 10.1016/j.cell.2012.03.037 22632969 PMC3383996

[12] G. Fusco , S. W. Chen , P. T. Williamson *et al.*, “ Structural basis of membrane disruption and cellular toxicity by α-synuclein oligomers,” Science 358(6369), 1440–1443 (2017). 10.1126/science.aan6160 29242346

[13] M. L. Choi , A. Chappard , B. P. Singh *et al.*, “ Pathological structural conversion of α-synuclein at the mitochondria induces neuronal toxicity,” Nat. Neurosci. 25(9), 1134–1148 (2022). 10.1038/s41593-022-01140-3 36042314 PMC9448679

[14] K. A. Conway , S. J. Lee , J. C. Rochet *et al.*, “ Acceleration of oligomerization, not fibrillization, is a shared property of both alpha-synuclein mutations linked to early-onset Parkinson's disease: implications for pathogenesis and therapy,” Proc. Natl. Acad. Sci. U. S. A. 97(2), 571–576 (2000). 10.1073/pnas.97.2.571 10639120 PMC15371

[15] K. M. Danzer , D. Haasen , A. R. Karow *et al.*, “ Different species of α-synuclein oligomers induce calcium influx and seeding,” J. Neurosci. 27(34), 9220–9232 (2007). 10.1523/JNEUROSCI.2617-07.2007 17715357 PMC6672196

[16] S. B. Prusiner , A. L. Woerman , D. A. Mordes *et al.*, “ Evidence for α-synuclein prions causing multiple system atrophy in humans with parkinsonism,” Proc. Natl. Acad. Sci. U. S. A. 112(38), E5308–E5317 (2015). 10.1073/pnas.1514475112 26324905 PMC4586853

[17] F.-X. Theillet , A. Binolfi , B. Bekei *et al.*, “ Structural disorder of monomeric α-synuclein persists in mammalian cells,” Nature 530(7588), 45–50 (2016). 10.1038/nature16531 26808899

[18] A. D. Stephens , M. Zacharopoulou , and G. S. K. Schierle , “ The cellular environment affects monomeric α-synuclein structure,” Trends Biochem. Sci. 44(5), 453–466 (2019). 10.1016/j.tibs.2018.11.005 30527975

[19] J. Burré , S. Vivona , J. Diao *et al.*, “ Properties of native brain α-synuclein,” Nature 498(7453), E4–E6 (2013). 10.1038/nature12125 23765500 PMC4255827

[20] B. Li , P. Ge , K. A. Murray *et al.*, “ Cryo-EM of full-length α-synuclein reveals fibril polymorphs with a common structural kernel,” Nat. Commun. 9(1), 3609 (2018). 10.1038/s41467-018-05971-2 30190461 PMC6127345

[21] R. Guerrero-Ferreira , N. M. Taylor , D. Mona *et al.*, “ Cryo-EM structure of alpha-synuclein fibrils,” elife 7, e36402 (2018). 10.7554/eLife.36402 29969391 PMC6092118

[22] Y. Li , C. Zhao , F. Luo *et al.*, “ Amyloid fibril structure of α-synuclein determined by cryo-electron microscopy,” Cell Res. 28(9), 897–903 (2018). 10.1038/s41422-018-0075-x 30065316 PMC6123497

[23] M. D. Tuttle , G. Comellas , A. J. Nieuwkoop *et al.*, “ Solid-state NMR structure of a pathogenic fibril of full-length human α-synuclein,” Nat. Struct. Mol. Biol. 23(5), 409–415 (2016). 10.1038/nsmb.3194 27018801 PMC5034296

[24] Y. Yang , Y. Shi , M. Schweighauser *et al.*, “ Structures of α-synuclein filaments from human brains with Lewy pathology,” Nature 610, 791–795 (2022). 10.1038/s41586-022-05319-3 36108674 PMC7613749

[25] B. I. Giasson , I. V. Murray , J. Q. Trojanowski *et al.*, “ A hydrophobic stretch of 12 amino acid residues in the middle of α-synuclein is essential for filament assembly,” J. Biol. Chem. 276(4), 2380–2386 (2001). 10.1074/jbc.M008919200 11060312

[26] J. A. Rodriguez , M. I. Ivanova , M. R. Sawaya *et al.*, “ Structure of the toxic core of α-synuclein from invisible crystals,” Nature 525(7570), 486–490 (2015). 10.1038/nature15368 26352473 PMC4791177

[27] C. Doherty , S. M. Ulamec , R. Maya-Martinez *et al.*, “ A short motif in the N-terminal region of α-synuclein is critical for both aggregation and function,” Nat. Struct. Mol. Biol. 27(3), 249–259 (2020). 10.1038/s41594-020-0384-x 32157247 PMC7100612

[28] A. D. Stephens , M. Zacharopoulou , R. Moons *et al.*, “ Extent of N-terminus exposure of monomeric alpha-synuclein determines its aggregation propensity,” Nat. Commun 11(1), 2820 (2020). 10.1038/s41467-020-16564-3 32499486 PMC7272411

[29] B. M. Burmann , J. A. Gerez , I. Matečko-Burmann *et al.*, “ α-synuclein regulation by chaperones in mammalian cells,” Nature 577(7788), 127 (2020). 10.1038/s41586-019-1808-9 31802003 PMC6930850

[30] T. S. Ulmer , A. Bax , N. B. Cole *et al.*, “ Structure and dynamics of micelle-bound human α-synuclein,” J. Biol. Chem. 280(10), 9595–9603 (2005). 10.1074/jbc.M411805200 15615727

[31] M. Schweighauser , Y. Shi , A. Tarutani *et al.*, “ Structures of α-synuclein filaments from multiple system atrophy,” Nature 585(7825), 464–469 (2020). 10.1038/s41586-020-2317-6 32461689 PMC7116528

[32] R. Cascella , S. W. Chen , A. Bigi *et al.*, “ The release of toxic oligomers from α-synuclein fibrils induces dysfunction in neuronal cells,” Nat. Commun. 12(1), 1814 (2021). 10.1038/s41467-021-21937-3 33753734 PMC7985515

[33] T. Bartels , J. G. Choi , and D. J. Selkoe , “ α-Synuclein occurs physiologically as a helically folded tetramer that resists aggregation,” Nature 477(7362), 107–110 (2011). 10.1038/nature10324 21841800 PMC3166366

[34] V. N. Uversky , J. Li , and A. L. Fink , “ Evidence for a partially folded intermediate in α-synuclein fibril formation,” J. Biol. Chem. 276(14), 10737–10744 (2001). 10.1074/jbc.M010907200 11152691

[35] C. Wang , C. Zhao , D. Li *et al.*, “ Versatile structures of α-synuclein,” Front. Mol. Neurosci. 9, 48 (2016). 10.3389/fnmol.2016.00048 27378848 PMC4913103

[36] A. Solanki , K. Neupane , and M. T. Woodside , “ Single-molecule force spectroscopy of rapidly fluctuating, marginally stable structures in the intrinsically disordered protein α-synuclein,” Phys. Rev. Lett. 112(15), 158103 (2014). 10.1103/PhysRevLett.112.158103 24785077

[37] A. S. Wentink , N. B. Nillegoda , J. Feufel *et al.*, “ Molecular dissection of amyloid disaggregation by human HSP70,” Nature 587(7834), 483–488 (2020). 10.1038/s41586-020-2904-6 33177717

[38] M. M. Dedmon , K. Lindorff-Larsen , J. Christodoulou *et al.*, “ Mapping long-range interactions in α-synuclein using spin-label NMR and ensemble molecular dynamics simulations,” J. Am. Chem. Soc. 127(2), 476–477 (2005). 10.1021/ja044834j 15643843

[39] C. W. Bertoncini , Y.-S. Jung , C. O. Fernandez *et al.*, “ Release of long-range tertiary interactions potentiates aggregation of natively unstructured α-synuclein,” Proc. Natl. Acad. Sci. U. S. A. 102(5), 1430–1435 (2005). 10.1073/pnas.0407146102 15671169 PMC547830

[40] A. C. M. Ferreon , Y. Gambin , E. A. Lemke *et al.*, “ Interplay of α-synuclein binding and conformational switching probed by single-molecule fluorescence,” Proc. Natl. Acad. Sci. U. S. A. 106(14), 5645–5650 (2009). 10.1073/pnas.0809232106 19293380 PMC2667048

[41] M. Sandal , F. Valle , I. Tessari *et al.*, “ Conformational equilibria in monomeric α-synuclein at the single-molecule level,” PLoS Biol. 6(1), e6 (2008). 10.1371/journal.pbio.0060006 18198943 PMC2174973

[42] A. C. M. Ferreon , C. R. Moran , J. C. Ferreon *et al.*, “ Alteration of the α-Synuclein folding landscape by a mutation related to Parkinson's disease,” Angew. Chem., Int. Ed. 49(20), 3469–3472 (2010). 10.1002/anie.201000378 PMC2972640 20544898

[43] H. Chen , G. Yuan , R. S. Winardhi *et al.*, “ Dynamics of equilibrium folding and unfolding transitions of titin immunoglobulin domain under constant forces,” J. Am. Chem. Soc. 137(10), 3540–3546 (2015). 10.1021/ja5119368 25726700 PMC4446711

[44] M. Zhao , D. Cascio , M. R. Sawaya *et al.*, “ Structures of segments of α‐synuclein fused to maltose‐binding protein suggest intermediate states during amyloid formation,” Protein Sci. 20(6), 996–1004 (2011). 10.1002/pro.630 21462277 PMC3104229

[45] S. Le , M. Yao , J. Chen *et al.*, “ Disturbance-free rapid solution exchange for magnetic tweezers single-molecule studies,” Nucl. Acids Res. 43(17), e113 (2015). 10.1093/nar/gkv554 26007651 PMC4787821

[46] X. Zhao , X. Zeng , C. Lu *et al.*, “ Studying the mechanical responses of proteins using magnetic tweezers,” Nanotechnology 28(41), 414002 (2017). 10.1088/1361-6528/aa837e 28766506

[47] M. Yu , Z. Zhao , Z. Chen *et al.*, “ Modulating mechanical stability of heterodimerization between engineered orthogonal helical domains,” Nat. Commun. 11(1), 4476 (2020). 10.1038/s41467-020-18323-w 32900995 PMC7479118

[48] A. Chappard , C. Leighton , R. S. Saleeb *et al.*, “ Single-molecule two-color coincidence detection of unlabeled alpha-synuclein aggregates,” Angew. Chem., Int. Ed. 62(15), e202216771 (2023). 10.1002/anie.202216771 PMC10946743 36762870

[49] S. Campioni , G. Carret , S. Jordens *et al.*, “ The presence of an air–water interface affects formation and elongation of α-synuclein fibrils,” J. Am. Chem. Soc. 136(7), 2866–2875 (2014). 10.1021/ja412105t 24460028

[50] J. Pujols , S. Peña-Díaz , I. Pallarès *et al.*, “ Chemical chaperones as novel drugs for Parkinson's disease,” Trends Mol. Med. 26(4), 408–421 (2020). 10.1016/j.molmed.2020.01.005 32277934

[51] J. Bieschke , J. Russ , R. P. Friedrich *et al.*, “ EGCG remodels mature α-synuclein and amyloid-β fibrils and reduces cellular toxicity,” Proc. Natl. Acad. Sci. U. S. A. 107(17), 7710–7715 (2010). 10.1073/pnas.0910723107 20385841 PMC2867908

[52] K. A. Conway , J.-C. Rochet , R. M. Bieganski *et al.*, “ Kinetic stabilization of the α-synuclein protofibril by a dopamine-α-synuclein adduct,” Science 294(5545), 1346–1349 (2001). 10.1126/science.1063522 11701929

[53] B. Ahmad and L. J. Lapidus , “ Curcumin prevents aggregation in α-synuclein by increasing reconfiguration rate,” J. Biol. Chem. 287(12), 9193–9199 (2012). 10.1074/jbc.M111.325548 22267729 PMC3308736

[54] J. Wagner , S. Ryazanov , A. Leonov *et al.*, “ Anle138b:A novel oligomer modulator for disease-modifying therapy of neurodegenerative diseases such as prion and Parkinson's disease,” Acta Neuropathol. 125(6), 795–813 (2013). 10.1007/s00401-013-1114-9 23604588 PMC3661926

[55] L. Tatenhorst , K. Eckermann , V. Dambeck *et al.*, “ Fasudil attenuates aggregation of α-synuclein in models of Parkinson's disease,” ACTA Neuropathol. Commun. 4(1), 39 (2016). 10.1186/s40478-016-0310-y 27101974 PMC4840958

[56] M. Zhu , S. Rajamani , J. Kaylor *et al.*, “ The flavonoid baicalein inhibits fibrillation of α-synuclein and disaggregates existing fibrils,” J. Biol. Chem. 279(26), 26846–26857 (2004). 10.1074/jbc.M403129200 15096521

[57] P. Robustelli , A. Ibanez-de-Opakua , C. Campbell-Bezat *et al.*, “ Molecular basis of small-molecule binding to α-synuclein,” J. Am. Chem. Soc. 144(6), 2501–2510 (2022). 10.1021/jacs.1c07591 35130691 PMC8855421

[58] L. L. Venda , S. J. Cragg , V. L. Buchman *et al.*, “ α-Synuclein and dopamine at the crossroads of Parkinson's disease,” Trends Neurosci. 33(12), 559–568 (2010). 10.1016/j.tins.2010.09.004 20961626 PMC3631137

[59] R. Petrosyan , S. Patra , N. Rezajooei *et al.*, “ Unfolded and intermediate states of PrP play a key role in the mechanism of action of an antiprion chaperone,” Proc. Natl. Acad. Sci. U. S. A. 118(9), e2010213118 (2021). 10.1073/pnas.2010213118 33619087 PMC7936342

[60] J. Lotharius and P. Brundin , “ Pathogenesis of Parkinson's disease: Dopamine, vesicles and α-synuclein,” Nat. Rev. Neurosci. 3(12), 932–942 (2002). 10.1038/nrn983 12461550

[61] S. W. Chen , S. Drakulic , E. Deas *et al.*, “ Structural characterization of toxic oligomers that are kinetically trapped during α-synuclein fibril formation,” Proc. Natl. Acad. Sci. U. S. A. 112(16), E1994–E2003 (2015). 10.1073/pnas.1421204112 25855634 PMC4413268

[62] L. Tosatto , M. H. Horrocks , A. J. Dear *et al.*, “ Single-molecule FRET studies on alpha-synuclein oligomerization of Parkinson's disease genetically related mutants,” Sci. Rep. 5(1), 16696 (2015). 10.1038/srep16696 26582456 PMC4652217

[63] M. L. Choi and S. Gandhi , “ Crucial role of protein oligomerization in the pathogenesis of Alzheime's and Parkinson's diseases,” FEBS J. 285(19), 3631–3644 (2018). 10.1111/febs.14587 29924502

[64] M. H. Ludtmann , P. R. Angelova , M. H. Horrocks *et al.*, “ α-synuclein oligomers interact with ATP synthase and open the permeability transition pore in Parkinson's disease,” Nat. Commun. 9(1), 2293 (2018). 10.1038/s41467-018-04422-2 29895861 PMC5997668

[65] R. Gaspar , G. Meisl , A. K. Buell *et al.*, “ Secondary nucleation of monomers on fibril surface dominates α-synuclein aggregation and provides autocatalytic amyloid amplification,” Q. Rev. Biophys. 50, e6 (2017). 10.1017/S0033583516000172 29233218

[66] K. Zhao , Y. Li , Z. Liu *et al.*, “ Parkinson's disease associated mutation E46K of α-synuclein triggers the formation of a distinct fibril structure,” Nat. Commun. 11(1), 2643 (2020). 10.1038/s41467-020-16386-3 32457390 PMC7250837

[67] W. Huang , S. Le , Y. Sun *et al.*, “ Mechanical stabilization of a bacterial adhesion complex,” J. Am. Chem. Soc. 144(37), 16808–16818 (2022). 10.1021/jacs.2c03961 36070862 PMC9501914

[68] H. Chen , H. Fu , X. Zhu *et al.*, “ Improved high-force magnetic tweezers for stretching and refolding of proteins and short DNA,” Biophys. J. 100(2), 517–523 (2011). 10.1016/j.bpj.2010.12.3700 21244848 PMC3325116

